# Comparing the Effects of Improvisation and Choreography on Older Adults with Mild Cognitive Impairment

**DOI:** 10.1093/geroni/igaf122.2734

**Published:** 2025-12-31

**Authors:** Yunjia Yang

**Affiliations:** University of Arizona, Tucson, Arizona, United States

## Abstract

This mixed-methods study compared the impact of improvisation and choreography on the cognition and well-being of older adults with MCI.

**Methods:**

20 participants with MCI were randomized into either the Improvisational Dance (ID) or Choreographic Dance (CD) group. Each group had two one-hour interventions per week for 10 weeks. Quantitative assessments on attention, memory, and creativity were done before and after the interventions. Participants completed the Positive Well-Being survey before and after the 1st, 5th, 10th, 15th, and 20th classes. Qualitative methods included focus group interviews, survey answers, and observations.

**Results:**

The ID group showed a significant improvement in creativity (p = .05), with greater gains compared to the CD group (p < .05). Both groups experienced a significant increase in short-term positive well-being after the 1-hour class (p < .001); however, only the CD group demonstrated significant long-term improvement over ten weeks (p < .05). Even though quantitative data did not show a statistically significant difference, mixed data showed the CD group presented a positive indication of improving attention/reaction time, visual-spatial short-term memory, physical benefits, and self-efficacy than the ID group. The ID group showed a positive indication of promoting social interaction and personal expression compared to the BP group. Qualitative results showed that self-efficacy, emotional engagement, expression, and social interactions contribute to Positive Well-being. Semi-structured improvisation in the ID group is key to promoting creativity. Remembering and repeating the movements with timing in the CD group are the potential key factors for improving attention, reaction time, and memory.

